# Role of the highly conserved G68 residue in the yeast phosphorelay protein Ypd1: implications for interactions between histidine phosphotransfer (HPt) and response regulator proteins

**DOI:** 10.1186/s12858-019-0104-5

**Published:** 2019-01-21

**Authors:** Emily N. Kennedy, Skyler D. Hebdon, Smita K. Menon, Clay A. Foster, Daniel M. Copeland, Qingping Xu, Fabiola Janiak-Spens, Ann H. West

**Affiliations:** 1 Department of Chemistry and Biochemistry, University of Oklahoma, Norman, OK 73019 USA; 2 Present Address: University of North Carolina, Chapel Hill, NC 27599 USA; 3 Present Address: Pacira Pharmaceuticals, San Diego, CA 92121 USA; 4 Present Address: GMCA at Advanced Photon Source, Argonne National Laboratory, Lemont, IL 60439 USA

**Keywords:** Two-component signal transduction, Ypd1, HPt proteins, Response regulator, Protein-protein interactions, Evolutionary conservation, Phosphotransfer

## Abstract

**Background:**

Many bacteria and certain eukaryotes utilize multi-step His-to-Asp phosphorelays for adaptive responses to their extracellular environments. Histidine phosphotransfer (HPt) proteins function as key components of these pathways. HPt proteins are genetically diverse, but share a common tertiary fold with conserved residues near the active site. A surface-exposed glycine at the *H + 4* position relative to the phosphorylatable histidine is found in a significant number of annotated HPt protein sequences. Previous reports demonstrated that substitutions at this position result in diminished phosphotransfer activity between HPt proteins and their cognate signaling partners.

**Results:**

We report the analysis of partner binding interactions and phosphotransfer activity of the prototypical HPt protein Ypd1 from *Saccharomyces cerevisiae* using a set of *H + 4* (G68) substituted proteins. Substitutions at this position with large, hydrophobic, or charged amino acids nearly abolished phospho-acceptance from the receiver domain of its upstream signaling partner, Sln1 (Sln1-R1). An in vitro binding assay indicated that G68 substitutions caused only modest decreases in affinity between Ypd1 and Sln1-R1, and these differences did not appear to be large enough to account for the observed decrease in phosphotransfer activity. The crystal structure of one of these *H + 4* mutants, Ypd1-G68Q, which exhibited a diminished ability to participate in phosphotransfer, shows a similar overall structure to that of wild-type. Molecular modelling suggests that the highly conserved active site residues within the receiver domain of Sln1 must undergo rearrangement to accommodate larger *H + 4* substitutions in Ypd1.

**Conclusions:**

Phosphotransfer reactions require precise arrangement of active site elements to align the donor-acceptor atoms and stabilize the transition state during the reaction. Any changes likely result in an inability to form a viable transition state during phosphotransfer. Our data suggest that the high degree of evolutionary conservation of residues with small side chains at the *H + 4* position in HPt proteins is required for optimal activity and that the presence of larger residues at the *H + 4* position would cause alterations in the positioning of active site residues in the partner response regulator.

**Electronic supplementary material:**

The online version of this article (10.1186/s12858-019-0104-5) contains supplementary material, which is available to authorized users.

## Background

Histidine phosphotransfer (HPt) proteins allow bacteria, yeast, and plants to expand beyond canonical two-component signaling pathways into multi-step phosphorelay pathways. These signaling proteins play crucial roles in regulating numerous cellular functions including those essential for growth and viability [1–4]. In bacteria, HPt domains are often additional domains embedded in histidine kinases of two-component signaling pathways [5–7]. In fungi and plants, HPts are typically stand-alone proteins that occupy branch points within multi-step His-to-Asp phosphorelays [8–11].

Multi-step phosphorelay systems consist of an upstream hybrid sensor histidine kinase (HHK), an HPt protein, and often multiple downstream response regulators (RR). Signal transduction is initiated when an external stimulus sensed by the HHK results in conformational changes that shift the protein from an inactive to an active conformation [12–14]. Phosphoryl transfer proceeds in a His-Asp-His-Asp manner, capitalizing on the chemical labilities of phospho-histidine and phospho-aspartate. Transfer of the γ-phosphate of adenosine triphosphate (ATP) occurs by autophosphorylation on a conserved histidine residue on the HHK, followed by transfer of the same phosphoryl group to a conserved aspartate residue on the receiver domain of the HHK. The HPt protein (or domain) acts as an intermediate in the signaling pathway by transferring the phosphoryl group from the HHK to a RR, which elicits a cellular response to the detected stress [1, 15, 16].

The Sln1 multi-step His-to-Asp phosphorelay of *Saccharomyces cerevisiae* has been extensively studied [17–20]. The pathway consists of the transmembrane HHK Sln1, the HPt Ypd1, and the RR proteins Ssk1 and Skn7 [11, 20–22]. According to the Saccharomyces Genome Database, Ypd1 is encoded by a single copy gene on chromosome IV, Ssk1 on chromosome XII, and Skn7 on chromosome VIII [23]. While Ypd1 has only a single upstream activator, it can phosphorylate both downstream RR proteins (Ssk1 and Skn7), depending on the encountered stress. Ssk1 is responsible for the osmotic stress response through the High Osmolarity Glycerol (HOG1) Mitogen Activated Protein Kinase (MAPK) pathway, while Skn7 is responsible for cell wall stress response and functions through direct transcriptional regulation of cell wall-related genes [24, 25]. Ypd1 is located in the cytoplasm of the cell, along with Ssk1, while Skn7 is sequestered in the nucleus [26]. Ypd1 readily translocates into the nucleus upon cell wall stress to phosphorylate Skn7 [26]. Under normal osmotic conditions, the Sln1-Ypd1-Ssk1 branch of the pathway is constitutively phosphorylated, and the downstream HOG1 pathway is repressed. Under hyperosmotic stress, Sln1 ceases to autophosphorylate, allowing dephosphorylated Ypd1 and Ssk1 to accumulate and activate the HOG1 pathway [27, 28]. The net result of HOG1 pathway activation is the production of intracellular glycerol that acts as a counter osmolyte [29, 30]. The Sln1 pathway is required for cell viability through regulation of the HOG1 pathway [11, 21, 31]. Defects in any step of the Sln1 pathway compromise the cell’s ability to respond to osmotic stress, leading to decreased cell viability [11, 27, 32].

Previous studies of the Sln1 pathway using yeast two-hybrid assays and X-ray crystallography demonstrated that Ypd1 interacts with the receiver domains of its response regulator binding partners using a common hydrophobic binding surface [33, 34]. All known structures of HPt proteins share a characteristic 4-helix bundle with a hydrophobic binding surface for RR binding [35–42]. The phosphorylatable histidine residue is located on the periphery of this hydrophobic patch on the αC helix of the 4-helix bundle and is completely solvent-exposed [1, 35, 41].

Despite their remarkably similar tertiary structure, HPt domains exhibit high sequence diversity. However, much of the αC helix and positions flanking the phosphorylatable histidine show moderate sequence conservation. These residues are often involved in maintaining structural integrity of the HPt protein or in forming intermolecular interactions with binding partners. Intramolecular interactions of conserved residues N61 and K67 of Ypd1 help stabilize the 4-helix bundle and pull the side chains away from the phospho-accepting histidine (H64). This ensures accessibility of the phosphoryl group for cognate response regulators [39, 41, 43]. In the BeF_3_^−^ bound complex of Ypd1 and Sln1-R1, the side chains of the Ypd1 K67 and R90 residues are involved in intermolecular interactions with the receiver domain [44, 45]. A glycine located four residues from the phosphorylatable histidine (*H + 4*) has been shown to be involved in protein-protein interactions and/or phosphoryl transfer in both yeast and bacteria [33, 45–48]. Previous results using a yeast two-hybrid assay indicated that substitution of a glutamine at the *H + 4* position in *S. cerevisiae* Ypd1(G68Q) may impair interactions with the receiver domains of its cognate RRs [33]. The ability of Ypd1-G68Q to both accept and transfer phosphoryl groups was also shown to be diminished [45, 46]. Further kinetic studies indicated that despite a dramatic decrease in the rate of phosphoryl transfer between Sln1-R1 and Ypd1-G68Q, binding affinity remained similar to wild-type (WT) [46]. We hypothesized that the glutamine substitution at residue 68 resulted in a catalytic defect rather than a significant change in protein binding affinity.

Here we report a comprehensive analysis of a more extensive set of substitutions at position 68 of Ypd1 to gain further insight into the evolutionary constraints leading to the unusually high conservation of glycine at this position. We combine bioinformatics, radio-isotopic phosphoryl transfer assays, X-ray crystallography, fluorescence-based binding data and molecular modelling techniques to highlight the importance of key intermolecular interactions required for optimal phosphotransfer between an HPt protein and its cognate RR.

## Results

### Residues in the *H + 4* position of HPt domains

**Fig. 1:**
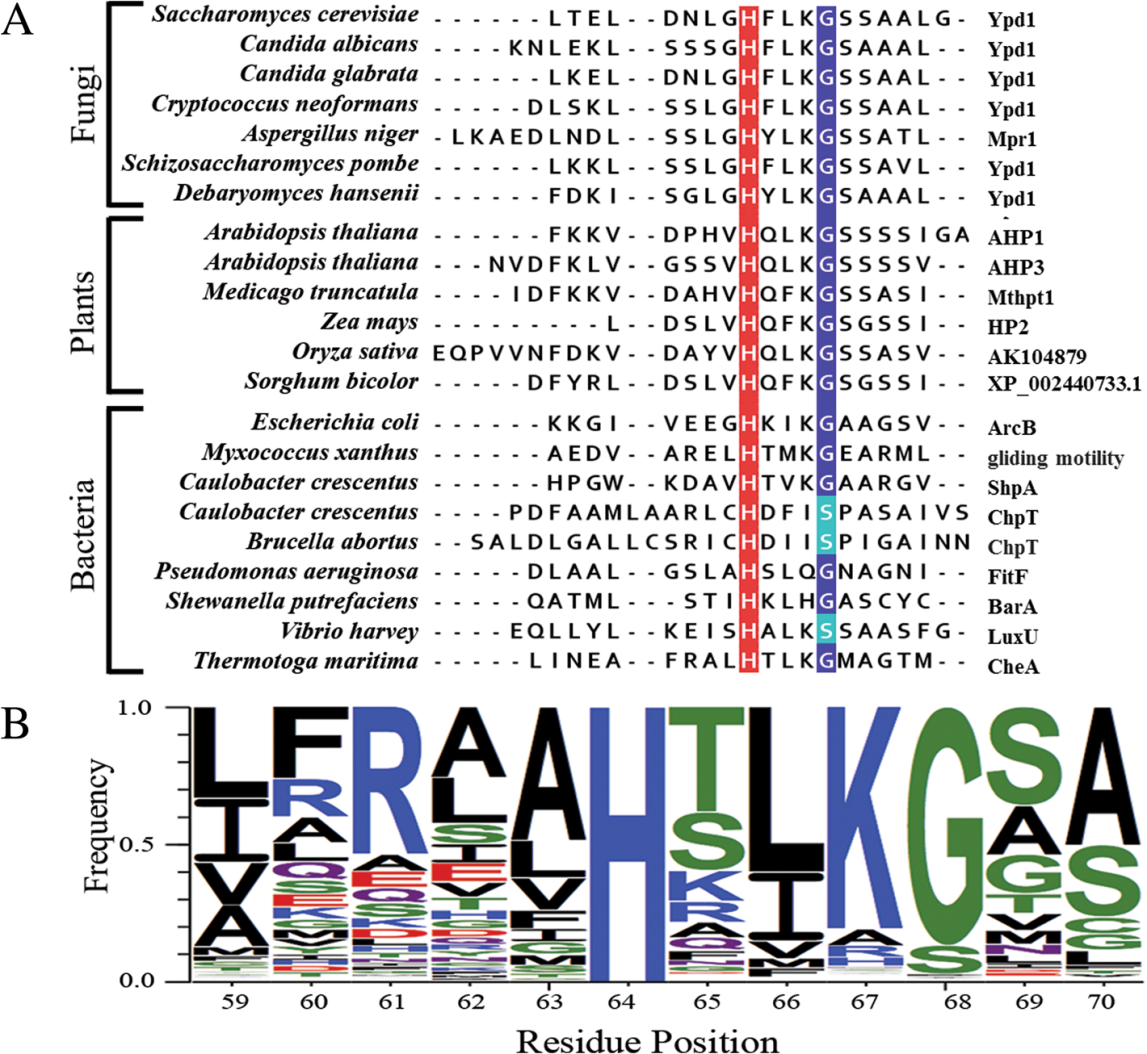
*H + 4* residue relative to the phosphorylatable histidine is highly conserved. **a** Representative sequence alignment of the αC region of HPt proteins/domains from fungi, plants, and bacteria. The phosphorylatable histidine residue is highlighted in red, and the *H + 4* residue is highlighted in blue. **b** Frequency of residues found in positions surrounding the phosphorylatable histidine are represented by a WebLogo [63] created using the complete sequence alignment. Indicated residue positions are based on the numbering of the *S. cerevisiae* Ypd1 HPt protein

Approximately 10,000 non-redundant sequences, representing bacteria, fungi, and plant HPt sequences obtained from the Pfam database [49], were analyzed to determine what percentage of residues were found in the *H + 4* position. Figure 1 shows the sequence alignment of the predicted αC and αD helices of the core 4-helix bundle from a representative set of HPt proteins and a WebLogo created using the complete alignment showing the frequency of each residue. The *H + 4* position is highly conserved throughout all sequences analyzed. Glycine was found in the *H + 4* position in approximately 87% of the sequences that were aligned, with approximately 10% of sequences having serine in the *H + 4* position (Table 1).

**Table 1:** Quantification of residues found in the *H + 4* position of HPt proteins/domains

Residue	Num. of Sequences	% of Total
G	8757	86.94
S	1051	10.43
P	98	0.97
A	76	0.75
T	31	0.31
N	28	0.28
H	12	0.12
E	6	0.06
D	4	0.04
R	3	0.03
V	2	0.02
I	1	0.01
C	1	0.01
L	1	0.01
Q	1	0.01
Y	1	0.01
W	0	0

### Non-conservative substitutions at the *H + 4* position disrupt phosphotransfer

**Fig. 2:**
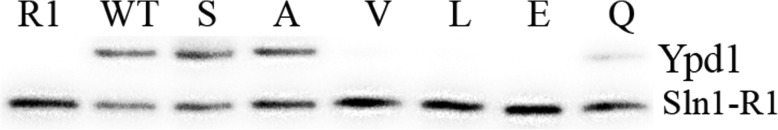
Ability of Ypd1 to accept phosphoryl groups is affected by nature of residue in the *H + 4* position. Phosphoryl transfer reactions contained equimolar concentrations of Sln1-R1 and Ypd1. Reaction was quenched at 5 min with stop buffer containing EDTA and separated by SDS-PAGE. The gel bands were detected by phosphorimaging and analyzed using ImageJ software. The amount of radiolabel in Ypd1-G68X mutants was quantified and compared with the amount of radiolabel seen for the band corresponding to wild-type YPD1 (normalized to 100%)

**Fig. 3:**
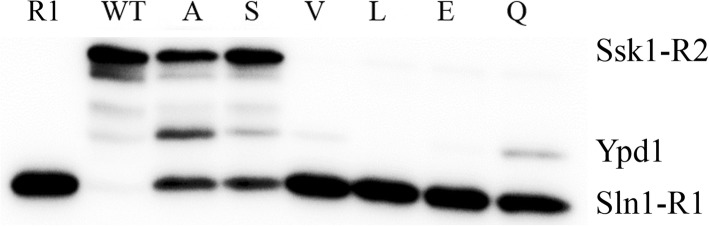
Residue in *H + 4* position alters ability of Ypd1 to function as a phosphorelay intermediate. Phosphoryl transfer reactions contained equimolar concentrations of Sln1-R1, Ypd1, and Ssk1-R2 proteins. Reactions were quenched at 5 min with stop buffer containing EDTA and separated by SDS-PAGE. The gel bands were detected by phosphorimaging and analyzed using ImageJ software. The amount of radiolabel in Ssk1-R2 bands in the presence of Ypd1-G68X mutants was quantified and compared with the amount of Ssk1-R2 radiolabel in the presence of wild-type Ypd1 (normalized to 100%)

Various amino acid residues (S, A, V, L, E, Q) were introduced to the G68 position of Ypd1 in order to analyze what effects amino acids other than glycine have on the phosphorelay activity of Ypd1. Phosphorelay experiments were performed using each of the Ypd1-G68X substituted proteins with the receiver domain of the upstream binding partner (Sln1-R1) as a donor and the receiver domain of the downstream binding partner (Ssk1-R2) as a recipient. Ypd1-G68S and Ypd1-G68A both accepted phosphoryl groups from Sln1-R1 at approximately the same level as wild-type Ypd1 (Table 2, Fig. 2). Ypd1-G68Q showed significantly decreased phosphorylation (~ 40% of wild-type) while Ypd1-G68 V, Ypd1-G68 L and Ypd1-G68E were severely impaired in accepting phosphoryl groups (less than 3% of wild-type) (Table 2, Fig. 2). In phosphorelay experiments (transfer from Sln1-R1 to Ypd1-G68X to Ssk1-R2) Ypd1-G68S and Ypd1-G68A exhibited phosphorelay activity at levels of ~ 80% to that of wild-type Ypd1. In contrast, Ypd1-G68Q showed severely impaired phosphorelay to Ssk1-R2 (Fig. 3), consistent with previously published results [45]. Due to their inability to accept phosphoryl groups from Sln1-R1, no phosphotransfer was observed from Ypd1-G68V, Ypd1-G68L or Ypd1-G68E to Ssk1-R2.

**Table 2:** Phosphorylation of Ypd1-G68X and phosphorelay activity

Protein	^a^Phosphorylation (%*)	^b^Phosphorelay (%*)
WT	100 ± 0	100 ± 0
G68S	94 ± 3.6	81 ± 18
G68A	82 ± 16	80 ± 8.0
G68V	2.5 ± 3.5	0.36 ± 0.6
G68L	0.4 ± 0.6	0.28 ± 0.5
G68E	0.3 ± 0.5	0.08 ± 0.15
G68Q	40 ± 8.3	0.9 ± 1.6

*Values are expressed as a percentage of wild-type. Standard deviations were calculated based on three replicates under similar conditions

^a^Phosphorylation of Ypd1 mutants from Sln1-R1 as phosphondonor

^b^Phosphorelay from Sln1-R1 to Ssk1-R2

### Substitutions at the *H + 4* position cause only modest changes in binding affinity

**Fig. 4:**
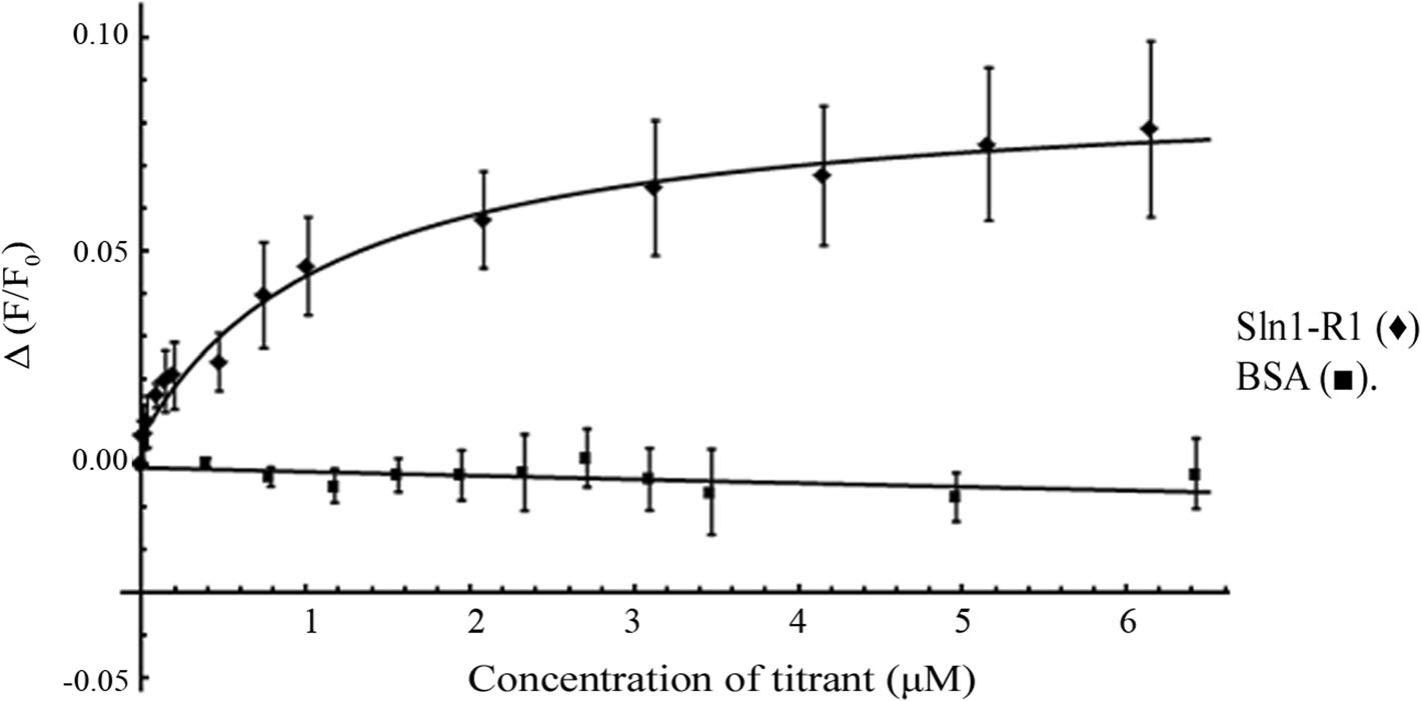
Saturation binding curve between Ypd1 and Sln1-R1 in a fluorescence binding assay. Fluorescence-based protein binding experiments using Ypd1-T12C~F titrated with Sln1-R1 (♦) or BSA (■). Fluoresceinated Ypd1-T12C (30 pmol) in 1.9 mL of reaction buffer was titrated with Sln1-R1 using a concentration range from 10 nM to 6 μM

A fluorescence-based in vitro binding assay was developed to gain insight into the differences in binding between Ypd1 and its partners. A substitution was made on Ypd1 (T12) adjacent to the hydrophobic binding patch (but in close proximity of the active site) to introduce a unique, solvent exposed cysteine. This cysteine was used for thiol-specific labelling with a fluorescent probe, 5-iodoacetamidofluorescein (5-IAF). Ypd1-T12C~F functions in vitro in phosphotransfer assays similarly to wild-type Ypd1 (data not shown). The titration of 5-IAF labelled Ypd1 with Sln1-R1 increased fluorescence intensity above baseline buffer dilutions, resulting in binding curves that appear to reach saturation (Fig. 4). Observed equilibrium dissociation constants (K_d_), calculated from these binding curves for this panel of mutant proteins, ranged from 0.5 to 3 μM as shown in Table 3. The observed K_d_ for the Sln1-R1 and Ypd1-T12C interaction, 0.94 ± 0.38 μM, is in agreement with the observed K_d_ between Sln1-R1 and wild-type Ypd1 calculated from published kinetics data [46, 50]. Though the estimated K_d_ values for the mutants were within the error of the K_d_ observed for wild-type Ypd1, substitutions with hydrophobic side chains showed a slight increase in K_d_ while hydrophilic side chains showed the opposite trend (Table 3).

**Table 3:** Observed dissociation constants (K_d_ in μM) for Ypd1 with Sln1-R1

WT	0.94 ± 0.38
G68V	2.9 ± 0.08
G68L	1.3 ± 0.09
G68E	0.5 ± 0.13
G68Q	0.6 ± 0.27

Binding constants and standard deviations were derived from three replicate titrations

### Structural integrity of the Ypd1 G68X mutants

**Fig. 5:**
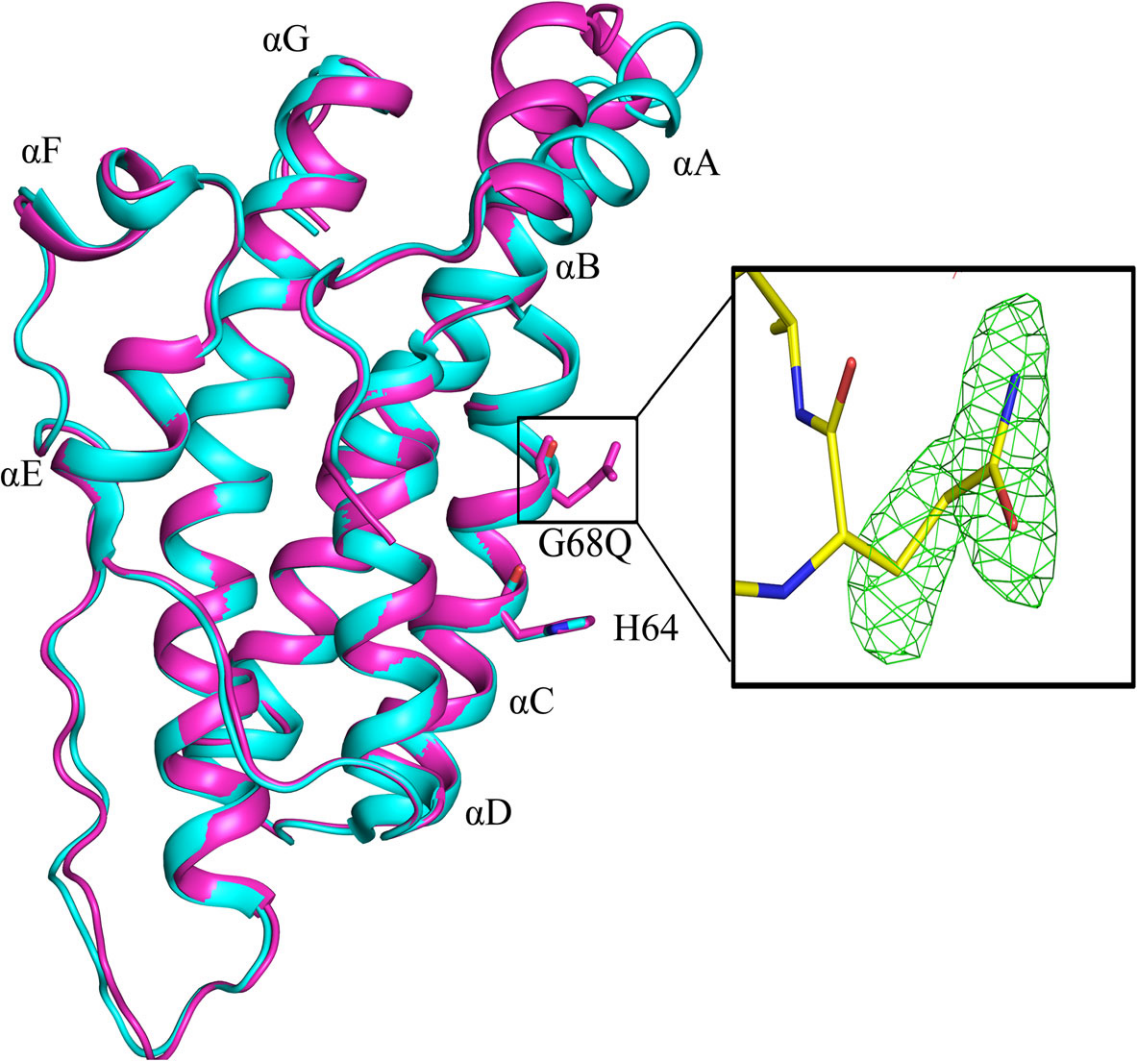
Ypd1-G68Q structure is similar to that of wild-type Ypd1. Overlay of wild-type Ypd1 (cyan) (PDB ID: 1QSP) and the Ypd1-G68Q mutant (magenta). The helices are numbered sequentially A to G from the N terminus to the C terminus, with the four-helix bundle core composed of helices B, C, D and G. The phosphorylatable histidine and *H + 4* glycine or glutamine are shown in stick representation. Movement of the αA helix is observed with a RMSD of 1.7 Å for this region. Inset: Electron density for the substituted Q residue at position 68 in Ypd1 as shown by the F_o_-F_c_ omit map (green mesh), contoured at 3.0 σ

**Fig. 6:**
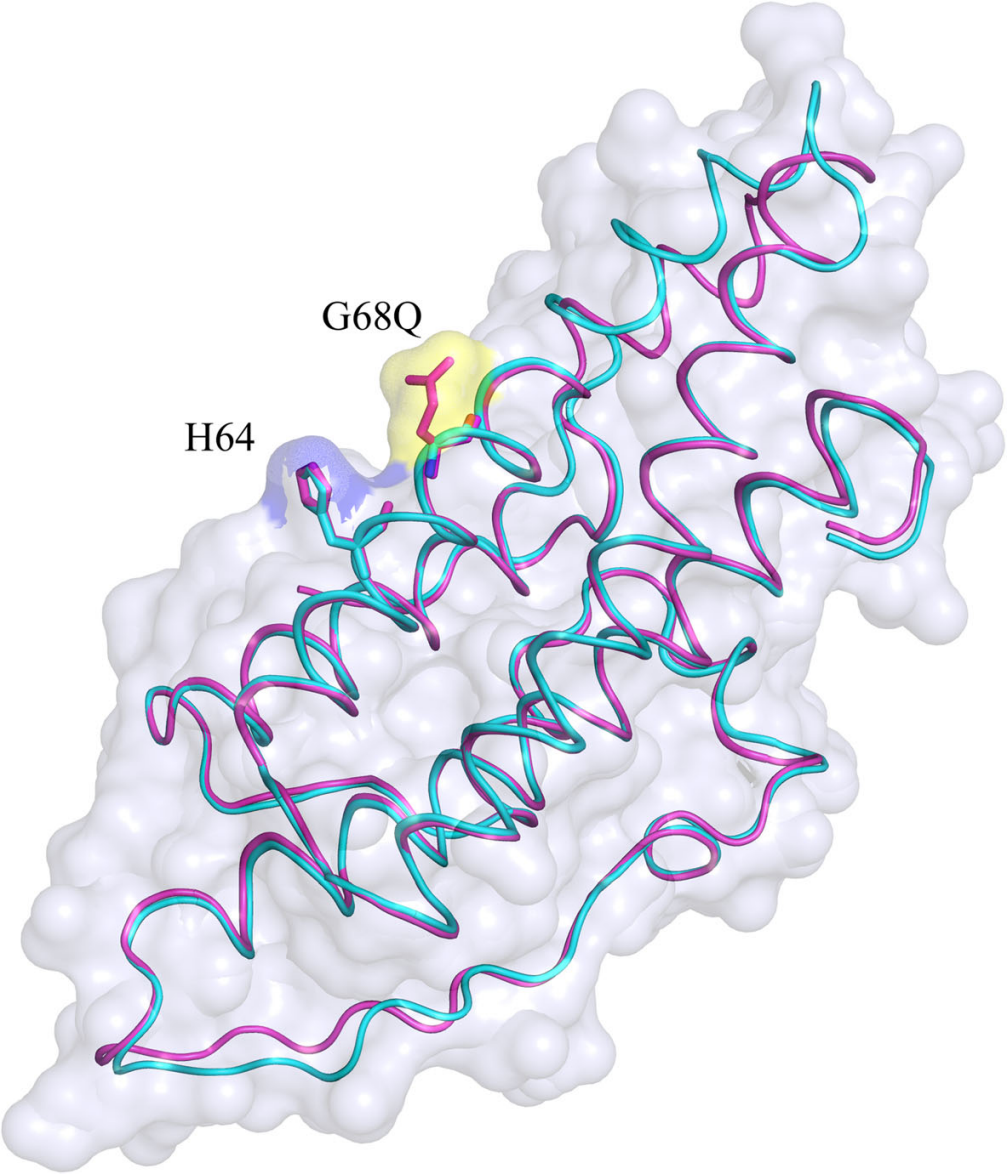
Glutamine residue adds bulk to the binding surface. Transparent surface of Ypd1-G68Q (magenta) overlaid with wild-type Ypd1 (cyan) (PDB ID: 1QSP). The volume of the H64 surface is shown in blue and the added bulk of the glutamine side chain is shown in yellow

The X-ray structure of the Ypd1-G68Q mutant was determined in order to ascertain structural integrity of the mutant and to gain insight into its loss of function characteristics. The Ypd1-G68Q mutant protein crystallized in space group P3_1_21. The structure was solved to a resolution of 1.98 Å using molecular replacement. The model contains residues 2–167 with an average B-factor of 23.73 Å^2^. X-ray data collection and refinement statistics are presented in Table 4. Clear electron density was observed for the side chain of the glutamine substitution at position 68 (see inset of Fig. 5). The root mean square deviation (RMSD) for all residues between the Ypd1-G68Q and wild type structure is 1.9 Å. Few differences are apparent when comparing this structure and the previously reported Ypd1 crystal structure (PDB ID: 1QSP), Figs. 5 and 6 [35, 44, 51]. The area around the site of phosphorylation is unperturbed with an RMSD of 0.2 Å for all residues in αC and αD helix. The N-terminal αA helix and the following turn (residues 11–21) show the largest shifts in the C-alpha positions with an RMSD of 1.7 Å. The αB helix is extended by one turn (residues 22–26) in the Ypd1-G68Q structure. The αA helix rotates approximately 30° around an axis at its N-terminus and αA helix makes a ¼ rotation around its longitudinal axis (Fig. 5). Upon the αA twisting movement, the originally exposed I13 and I17 become buried, while L14 and I18 are now exposed. Thus, hydrophobic interactions between αA and the rest 4-helix bundle are maintained, but by different residues. Minor shifts in the orientations of exposed side chains account for the remainder of the deviation between Ypd1 structures. The crystal structure was also determined for Ypd1-G68E (non-functional). The structure showed little overall change in comparison to wild-type Ypd1 (data not shown). In order to assess structural integrity of other Ypd1 variants, Fourier-transform infrared spectroscopy (FTIR) data were collected for wild-type Ypd1, Ypd1-G68S (functional) and Ypd1-G68L (non-functional). Despite some sample-to-sample variation, the FTIR spectra of tested Ypd1 variants were consistent with the observed well-folded α-helical nature of HPt proteins (data not shown).

**Table 4:** Data collection and Refinement Statistics for Ypd1-G68Q

Data Collection	
Space group	P3_1_21
Unit cell dimensions (Å, °)	a = b = 76.7, c = 66.7 and α = β = 90, γ = 120
Resolution range (Å)	38.36–1.98 (2.051–1.98)^a^
Total number of reflections	100,588
Number of unique reflections	15,936 (1400)
Average redundancy	
% completeness	97 (72)
R_merge_ (%)^b^	0.051
CC_1/2_^c^	0.879
Mean I/σI	31 (1.95)
Refinement Statistics	
Resolution Range (Å)	38.36–1.98 (2.051–1.98)
*R*_work_ (%)^d^	17.8
R_free_ (%)^e^	20.7
Average B-factor (Å^2^)	23.73
# of Protein Atoms	1363
# of Waters	166
RMSD bond length (Å)	0.010
RMSD angles (°)	1.0
Ramachandran plot (%)	
Most favored	98.78
Additionally allowed	1.2
Disallowed	0

^a^Values in () are for the highest resolution shell

^b^R_merge_ = Σ(I - 〈I〉)I/Σ(I), where I is the intensity measurement of a given reflection and 〈I〉 is the average intensity for multiple measurements of this reflection

^c^Half-set correlation coefficient CC1/2 as defined in Karplus and Diederichs [78]

^d^R_work_ = Σ||F_o_| - |F_c_|| / Σ|F_o_|, where F_o_ and F_c_ are the observed and calculated structure factors respectively

^e^R_free_ was calculated with 5% of the diffraction data that were selected randomly and not used throughout refinement

### Molecular modelling of *H + 4* substitutions

To further explain the effects of the *H + 4* residue substitutions in Ypd1, residues were modelled onto the existing Sln1-R1/Ypd1-BeF_3_^−^ structure (PDB ID: 2R25). Brief energy minimization and relaxation steps were performed on the mutant active sites to remove clashes and estimate possible localized perturbations caused by each substitution. The following Ypd1-G68 variants were tested, in addition to a wild-type control: S, V, E, L and Q. The wild-type Ypd1 model showed little structural perturbation upon relaxation, largely preserving the original phosphoryl alignment and interaction network. Ypd1-G68L also exhibited relatively little perturbation, though the leucine side chain was arranged such as to hinder movement of the highly conserved K1195 (see Additional file 1: Figure S1 for active site models of Ypd1-G68X mutant proteins in complex with P~Sln1-R1). The Ypd1-G68V model suggested distortion of the phosphoryl alignment, increasing the distance between A1174 on the β4α4 loop of Sln1-R1 and the closest phosphoryl oxygen. Like in Ypd1-G68L, the substituted valine side chain was oriented towards K1195 in Sln1. The Ypd1-G68E model exhibited similar effects and showed a larger shift in A1174 on the β4α4 loop of Sln1-R1. The positioning of the acidic side chain suggests an ability to form an intramolecular salt-bridge with K67 of Ypd1. While the Ypd1-G68S model showed an increase in the distance to A1174, the serine side chain was able to directly interact with the phosphoryl group. Finally, the Ypd1-G68Q model exhibited perturbations resembling the Ypd1-G68V model. However, despite sharing similarities with the hydrophobic substitution, the mutant Ypd1-G68Q side chain is oriented away from the phosphorylatable H64 and appeared capable of forming a hydrogen bond with residues outside the active site. The hydrophilicity of the glutamine residue, along with this reorientation, may reduce potential clashes with the K1195 side chain and allow for the low-level functionality of Ypd1-G68Q. The additional intermolecular interactions seen in the Ypd1-G68Q and Ypd1-G68E models are likely responsible for the modest increases in binding affinity observed with these mutants (Table 4). While these modelling observations support the mutant phosphotransfer data, the limitations of static structural models suggest that more extensive in silico studies are needed.

## Discussion

HPt proteins play critical roles in signal transduction. Their primary function is to transfer phosphoryl groups from the receiver domain of a HHK to the receiver domain of an RR protein. Bacteria and yeast with deleterious mutations of HPt genes are either inviable or exhibit decreased cell viability through suboptimal pathway regulation [9, 11, 52–55]. For example, the HPt domain of the HK-RR-HPt protein ArcB from *E. coli* transmits a signal to the terminal response regulator ArcA. Mutants of residues in ArcB^C^, notably the *H + 4* residue G721, show a decrease in phosphotransfer [47]. Similarly, substitution of the *H + 4* residue (G52) of the P1 domain from CheA (an HPt domain) has a detrimental effect on chemotaxis [48]. The Ypd1-G68Q mutant from *S. cerevisiae* showed significantly diminished in vitro phosphorylation from its upstream donor Sln1 and no observable phosphotransfer to the downstream acceptor Ssk1 [45]. In this study, we aimed to investigate the role of this *H + 4* residue in His-to-Asp multi-step phosphorelay systems.

### Small residues are highly conserved at the *H + 4* position

Because *H + 4* substitutions in HPt domains result in detrimental effects in both bacteria and yeast, a comprehensive sequence alignment was performed on all protein sequences predicted to be stand-alone HPt proteins or multi-domain proteins with embedded HPt domains. Residues with small-volume side chains were found to occupy the *H + 4* position in 98% of the sequences analyzed. Glycine appears in ~ 87% of cases, while ~ 10% were found to have serine and ~ 1% were found to have alanine. Such high conservation suggests that the presence of a small residue is essential for HPt domain function, including proper interaction with cognate binding partners and phosphotransfer activity.

### Substitutions at the *H + 4* position do not alter the overall structure of Ypd1

To test if the functional defects observed for the *S. cerevisiae* Ypd1-G68X substitutions were a result of loss of structural integrity, we used X-ray crystallography to determine the structure of the Ypd1-G68Q mutant. We did not observe any broad overall structural changes, with the exception of movement of the αA helix. Electron density confirmed the presence of the bulkier Gln side chain at position 68 (Figs. 5 and 6). The intrinsic flexibility of αA suggests that the observed movement is likely due to crystal packing and not a static structural alteration [41]. A crystal structure was also determined for Ypd1-G68E, and FTIR data were collected for Ypd1-G68S and Ypd1-G68L, all of which confirmed proper folding of the proteins (data not shown). As no overall changes were observed, we hypothesized that the *H + 4* glycine must be important for either phosphotransfer and/or protein-protein interactions.

### Diminished function as a phosphorelay protein is not a result of impaired binding affinity

By creating a series of amino acid substitutions in the *H + 4* position, we determined that substitutions of smaller residues (S, A) at the *H + 4* position still allowed for phosphotransfer at near wild-type level. Substitutions introducing larger side chain volumes (V, L, E) drastically inhibited the ability of Ypd1 to serve as both an acceptor and donor of phosphoryl groups. The only exception was the Ypd1-G68Q substitution, where a large hydrophilic side chain still allowed for partial phosphotransfer activity. In vitro fluorescence-based binding experiments between Sln1-R1 and Ypd1-G68X mutants showed no substantial change in binding affinity as a consequence of the substitutions. These data demonstrate that the diminished phosphotransfer activity that was observed for the larger side chain substitutions is likely not a consequence of changes in HPt:RR binding affinity. Taken together, our results suggest that interface perturbations introduced by the non-functional mutants at position 68 are negatively affecting catalysis of the phosphotransfer reaction.

### Substitutions at the G68 position perturb active site residues involved in catalysis

**Fig. 7:**
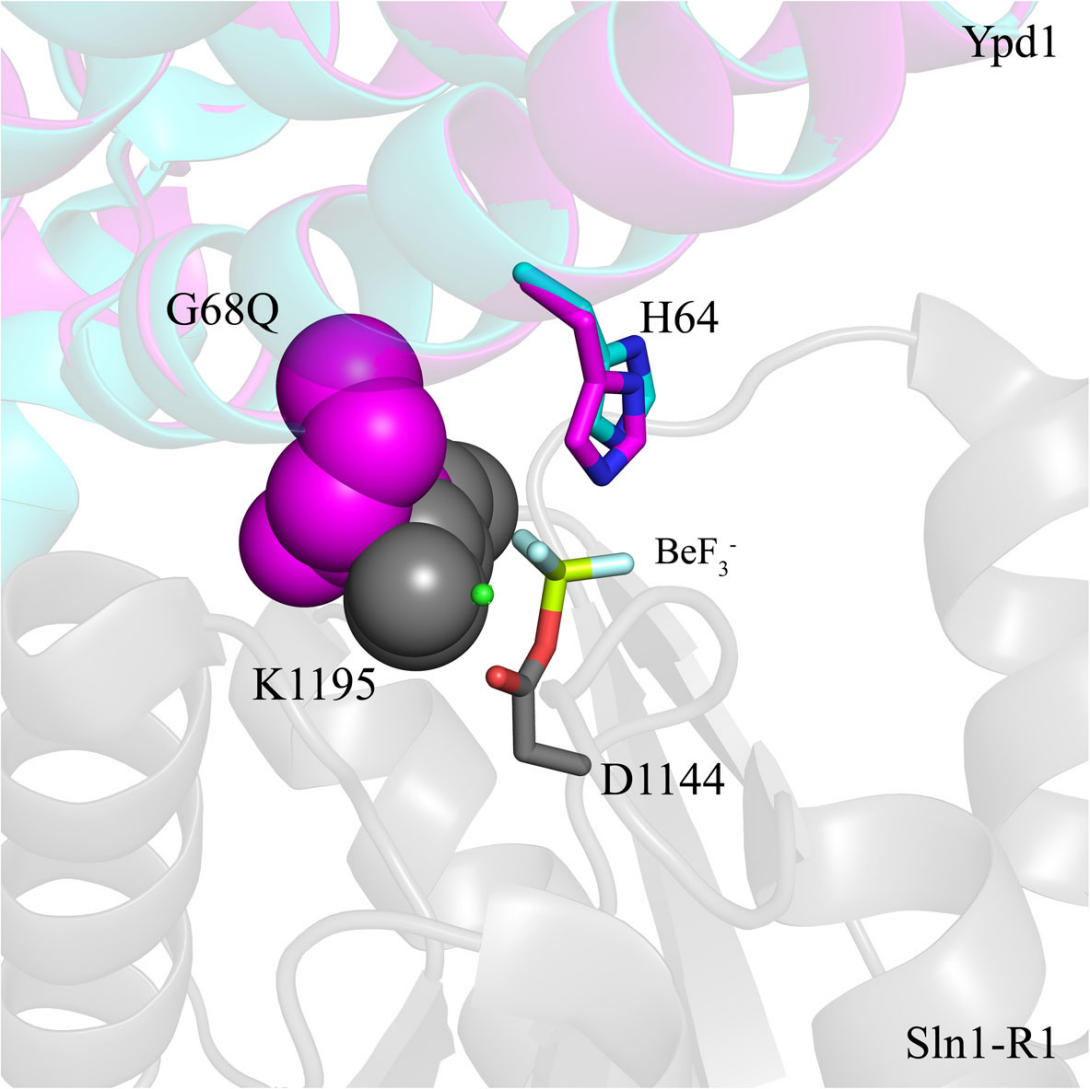
Clashes between Ypd1-G68Q and Sln1-R1. Overlay of the Ypd1-G68Q mutant (magenta) with the Ypd1•Sln1-R1•Mg^2+^•BeF_3_^−^ complex (cyan) (PDB ID: 2R25). The G68Q substitution of Ypd1 occupies the same space as the K1195 residue of Sln1-R1 (gray). G68Q and K1195 are shown in space filling representation. Bound Mg^2+^ is colored green and BeF_3_^−^ is shown in stick representation

Our lab previously showed that there is a hydrophobic docking site on the surface of Ypd1 for all three cognate binding partners. It consists of regions of the αA, the N-terminus of αB and the C-terminus of αC helices of Ypd1 [33]. From the crystal structures of Sln1-R1 in complex with Ypd1, we noted that the interfaces are highly complementary. The small volume of the response regulator active site cavity would not easily accommodate a bulky side chain such as G68Q in Ypd1. If this additional bulk does not inhibit binding, as our fluorescence-based binding data confirms, it must be interfering with key residues involved in the phosphotransfer reaction. When the structures of Ypd1-G68Q and Ypd1 from the BeF_3_^−^ activated complex (PDB ID: 2R25) are aligned, the mutant glutamine side chain clashes with the highly conserved K1195 residue of Sln1-R1 (Fig. 7). K1195 has been implicated in the stabilization of the transition state formed during the His-Asp phosphotransfer event [56]. Any changes to its ability to neutralize the negatively charged transition state during the reaction may have a significant effect on phosphotransfer between Sln1-R1 and Ypd1.

Molecular modelling suggests that the side chains of Ypd1-G68V and Ypd1-G68L remain in close proximity to the K1195, likely hindering its ability to move and affecting this stabilization role. Additionally, several other factors may be affected by the substitutions. Previous work found that a conserved in-line orientation is critical for the nucleophilic attack and phosphotransfer between the histidine and aspartate residues [57]. Correct geometry is maintained for HPt proteins and their RRs by a narrow channel through which the histidine can access the aspartate. In the Sln1-R1/Ypd1 complex, this is formed by adjacent residues on both proteins, including highly conserved positions such as Q1146, T1173, A1174 and K1195 on Sln1-R1, and K67, G68 and Q86 on Ypd1. Disruption to the arrangement of these residues would adversely affect the phosphotransfer reaction. Models of the V, E and Q substitutions exhibited various levels of distortion in this arrangement. The likely explanation for this is the introduction of a large chemical group into the active site causes steric clashes, though more extensive in silico studies are needed to confirm the full effects. Clashes may affect interactions with the β4α4 loop on Sln1-R1, particularly with the G68E substitution.

Upon phosphorylation, Sln1-R1 undergoes a conformational change involving the β4α4 region. This allows for the transfer channel to form, and for T1173 (side chain) and A1174 (main chain) to hydrogen bond with the oxygen atoms on either side of the tetrahedral phosphoryl group likely serving to stabilize the negatively charged pentavalent transition state during transfer. Modelling suggests that the larger substitutions (V, E and Q) alter the interactions of this region, increasing the distance between A1174 and the flanking phosphoryl oxygen atom. While the Ypd1-G68Q and Ypd1-G68S models also exhibited this effect, the hydrogen bonding ability of the glutamine/serine side chains produce different results. The glutamine side chain in Ypd1 is oriented away from the key active site residues in Sln1. This likely makes it more favorable and/or stable than a large hydrophobic side chain within the highly charged active site, however, we cannot rule out the possibility of changes in orientation during complex formation that may not be detectable in the models. The additional hydrogen bonding ability may also explain the increase in observed binding affinity between Ypd1-G68Q and Sln1-R1. The serine side chain was able to form a hydrogen bond directly with the phosphoryl group, compensating for the alteration in bonding with A1174. This may explain why serine is the second most abundant residue found at the *H + 4* position in HPt proteins.

### Implications for histidine kinases

Interestingly, while both HPt and HK domains interact with receiver domains using phosphorylatable histidine residues, the strict *H + 4* conservation found for HPt domains does not translate to the dimerization and histidine phosphotransfer domain (DHp) of HK proteins, even though this domain maintains a 4-α-helical bundle for interaction with receiver domains of RRs. In most HKs, a threonine or asparagine occupies the *H + 4* position in the conserved H-box motif of the DHp domain [58–60]. The *H + 4* position has also been implicated as a crucial residue for determining whether a HK will have phosphatase activity. In the osmosensor EnvZ, an *H + 4* threonine was crucial for activity [61]. Comparison of HK:RR structures HK853:RR468 (PDB ID: 3DGE) and ThkA:TrrA from *Thermotoga maritima* (PDB ID: 3A0R) with HPt:RR structures Sln1-R1: Ypd1 (PDB ID: 1OXB) and CheA-P1:CheY (PDB ID: 2LP4) demonstrates that the protein-protein interactions occur in different regions on the RR partner. RR proteins interact with HK proteins using primarily the β3α3/β4α4 regions, but interact with HPt proteins using the β1α1/β2α2/β3α3 regions. An altered domain orientation allows for the larger residues found near the phosphorylatable histidine on most HKs. Large residues on the surface of the HPt proteins in the *H + 4* location, as we have shown through radio-isotopic phosphotransfer assays and molecular modelling, affect residues crucial for proper HPt:RR interactions and phosphotransfer. Additional structural complexes of HK:RR cognate pairs as well as HPt:RR pairs would help to further support this observation.

## Conclusions

In this study, we created and characterized a series of residue substitutions at G68 in the *S. cerevisiae* HPt protein, Ypd1, to demonstrate the necessity of a small residue in this position for proper HPt function. These results help to explain the high sequence conservation observed at this position in all known HPt proteins/domains. We subsequently found that even with large, hydrophobic substitutions, the observed binding affinity between Ypd1 and its upstream partner Sln1-R1 was unaffected. The binding data suggested that diminished phosphotransfer activity was more likely due to interference in catalysis. Structural analysis using the crystal structure of the partially functional Ypd1-G68Q mutant and comparison with known crystal structures showed that the G68 substitutions could be interfering with key active site residues. We used molecular modelling to visualize the potential effects on these catalytic residues as a result of the introduction of larger side chains into the active site. These observations suggest how the larger side chains (G68V, G68L, G68E) abolish phosphotransfer, while Ypd1-G68Q is still partially functional. In summary, the presence of a highly-conserved glycine near the site of phosphorylation is sterically and mechanistically significant and essential for phosphotransfer between HPt proteins and cognate response regulator binding partners.

## Methods

### Sequence alignment

The full alignment of the HPt family (entry PF01627) was downloaded from the Pfam database, including sequences of both stand-alone HPt proteins and HK proteins with HPt domains [49]. This sequence list was edited with Jalview [62] to remove redundancy (with a 98% identity threshold). Any sequence lacking a conserved phosphorylatable histidine was removed. The number of non-redundant sequences remaining was approximately 10,000. Sequences were sorted and counted according to the residue at the *H + 4* position. Jalview was used to construct a representative sequence alignment. WebLogo3 was used to create a sequence logo from the full alignment [63].

### Expression and protein purification

A pUC-Ypd1 plasmid was expressed in *Escherichia coli* and the protein was purified using ammonium sulfate precipitation, anion exchange chromatography and size exclusion chromatography, as described previously [43]. The pUC-Ypd1 plasmid was then used as a template to create all Ypd1-G68X mutants using site-directed mutagenesis PCR. Proteins were expressed in DH5α *E. coli* cells and protein purification was performed similarly to wild-type Ypd1.

The *YPD1* gene was also inserted into a pET21a (+) vector for expression using the T7 promotor. To covalently attach a fluorescein molecule near the active site of Ypd1, T12 was mutated to a cysteine residue (pET-Ypd1-T12C) through two-step PCR site-directed mutagenesis. The resulting plasmid was used as a template for the creation of the double mutants. Various amino acid residues (S, A, V, L, E, Q) were introduced to the G68 position. Proteins were expressed in BL21(DE3) Star cells and purified similarly to wild-type Ypd1.

The expression vector encoding the Sln1 kinase domain (GST-Sln1-HK), was kindly provided by R. Deschenes (University of South Florida). GST-Sln1-HK was expressed and purified as a glutathione S-transferase (GST) fusion protein using glutathione Sepharose 4B resin. Protein was stored and used in assays in bead-bound form, as previously described [22].

The receiver domain from Sln1 (Sln1-R1) and the receiver domain of Ssk1 (Ssk1-R2) were inserted into a pFJS16 vector, as previously described [64]. These proteins were expressed in *E. coli* Rosetta (DE3) cells (Novagen) as a fusion protein containing C-terminal intein and chitin binding domains and were purified by chitin affinity chromatography and gel filtration, as described previously [22, 64].

### Crystallization

Hexagonal form Ypd1-G68Q mutant crystals were grown using an initial protein concentration of 10 mg/ml equilibrated in hanging drops over a reservoir of 0.1 M sodium acetate, pH 5.0, 0.2 M ammonium acetate and 25–30% PEG 4000 (Fluka, 81,242).

### X-ray data collection and processing

Data were collected at the OU Macromolecular Crystallography Lab using CuKα radiation (1.54 Å wavelength) at 100 K by flash freezing crystals in liquid N_2_. The data were recorded with a Dectris Pilatus P200K hybrid pixel detector using oscillations of 0.5° and processed using HKL2000 to a resolution of 1.98 Å [65]. The space group was determined to be P3_1_21 with unit cell parameters of *a* = *b* = 76.7 Å, *c* = 66.7 Å α = β = 90°, γ = 120°. One molecule was found in the asymmetric unit. Data processing and refinement statistics are presented in Table 4.

### Structure solution and refinement

Molecular replacement was performed using Phaser from the Phenix suite of programs [66–68] to determine the initial phases for the Ypd1-G68Q structure. The structure of wild-type Ypd1 (PDB ID: 1QSP) was used as a template for molecular replacement [43]. Refinement was done using Phenix-refine [69]. Clear difference electron density was observed for the glutamine side chain at position 68 in an F_o_-F_c_ electron density map. Coot [70] was used for model adjustments after each cycle of refinement. The final R_work_ was 17.8% with an R_free_ of 20.7%. Coordinates for the Ypd1-G68Q structure were deposited to the Protein Data Bank (PDB ID: 6M7W).

### Phosphorelay assay

The Sln1-HK domain bound to glutathione-Sepharose 4B resin was phosphorylated via incubation with 0.66 μM [γ-^32^P]-ATP (3000 Ci/mmol) for 30 min according to previously published protocols [19]. Unincorporated [γ-^32^P]-ATP was washed from phospho-Sln1-HK with 50 mM Tris-HCl, pH 8.0, 100 mM KCl, 15 mM MgCl_2_, 2 mM DTT, and 20% glycerol by three consecutive centrifugations (1 min at 1000 g). Sln1-R1 was added to the reaction and incubated for 5 min with phospho-Sln1-HK. Sln1-HK was removed from the reaction through gentle centrifugation, leaving only phosphorylated Sln1-R1 in solution. Phospho-Sln1-R1 was then used as a donor to phosphorylate all Ypd1 proteins. Ypd1 proteins were phosphorylated by incubation with an equimolar concentration of Sln1-R1 for 5 min. To test the ability of the Ypd1-G68X mutants to transfer the phosphoryl group to downstream binding partners, Ypd1~P was incubated with an equimolar concentration of Ssk1-R2 and an aliquot was obtained at 5 min. The reaction was quenched with stop buffer containing EDTA and samples were separated on a 15% SDS polyacrylamide gel and electrophoresed at 200 V for 45 min for visualization. The SDS gels were wrapped in plastic wrap and exposed to a phosphorimager screen. The radioactivity of each band was quantified using a Typhoon phosphorimager (Molecular Dynamics). Band intensities were quantified using ImageJ [71].

### Fluorescence binding assay

A substitution was made to Ypd1-T12 to introduce a unique, solvent-exposed cysteine for thiol-specific labelling with a fluorescent probe. Purified Ypd1-T12C and Ypd1-T12C-G68X mutants were buffer-exchanged into 50 mM potassium phosphate, pH 9.0 and 1 mM β-mercaptoethanol. Proteins were incubated in darkness for 2 h at room temperature with a 7-fold molar excess of 5-IAF for covalent labelling of Ypd1-T12C. Unincorporated 5-IAF was removed by exchanging labelled Ypd1 proteins into 20 mM Tris, pH 8.0, 50 mM NaCl and 10 mM MgCl_2_ using a GE HiTrap desalting column. The concentration of fluorescein bound to protein was estimated by absorption at 492 nm, and protein concentration was estimated by BioRad protein assay revealing that 70–90% of the Ypd1 molecules were labelled. Fluorescently-labelled proteins were aliquoted and stored in the presence of 10% glycerol at − 20 °C.

The Sln1-R1 receiver domain was purified as described previously, however, fluorescence reaction buffer (20 mM Tris, pH 8.0, 50 mM NaCl and 10 mM MgCl_2_) was substituted during size exclusion chromatography. Chelex® resin (BioRad) was used to strip contaminating cations from the Tris-salt solution before magnesium chloride was added to the buffer.

Binding of Sln1-R1 to fluorescein labelled Ypd1-T12C induces a change in the fluorescein moiety resulting in altered fluorescence intensity. A Fluoromax 4 Spectrofluorometer from Horiba Scientific, temperature controlled to 23.0 °C, was used to observe changes in fluorescence caused by binding. IAF-labelled Ypd1-T12C (30 pmol) was diluted to 1.9 mL in fluorescence reaction buffer, and Sln1-R1 was titrated into the reaction such that the concentration in the cuvette ranged from 10 nM to 6 μM. Upon addition of Sln1-R1, the solution was mixed with a magnetic stir bar for 30 s and allowed to rest for an additional 20 s before reading fluorescence intensity with absorbance at 492 nm and emission at 515 nm. Intensity after each addition (F) as a fraction of intensity from labelled Ypd1 alone (F_0_) was calculated for titration with Sln1-R1 and buffer alone. The difference between these two normalized intensities (F/F_0_, Sln1-R1 – F/F_0_, buffer) indicates binding of Sln1-R1 to labelled Ypd1. Plotting change in fluorescence intensity caused by Sln1-R1 versus concentration of Sln1-R1 shows a binding curve with saturation at high concentrations. These curves were fitted using Mathematica [72] to an expanded quadratic equation with parameters accounting for fluorescence from both the bound and unbound states of Ypd1 and the dissociation constant for the Ypd1:Sln1-R1 complex was calculated. The average dissociation constant and standard deviation of the mean are reported here.

### Molecular modelling

Ypd1-G68X substitutions were modelled initially in PyMOL. For Ypd1-G68Q and Ypd1-G68E (unpublished data), crystal structures were aligned to the existing Sln1-R1/Ypd1-BeF_3_^−^ complex (PDB ID: 2R25). The mutant Ypd1 and Sln1-R1 molecules were extracted for brief energy minimization and relaxation. For the remaining substitutions, residues were modelled directly into the active site of the Sln1-R1/Ypd1-BeF_3_^−^ structure. In each model, the BeF_3_^−^ ligand atoms were replaced with a phosphoryl group. Each system was stripped of crystallographic waters and ligands, with only the Mg^2+^ cation being retained. Structures were then re-solvated in an orthorhombic solvent box with 10 Å padding using the TIP3P solvent model [73]. Finally, systems were neutralized and ionized to a concentration of 0.15 M NaCl. System preparation was done using the Maestro graphic user interface from the Schrödinger molecular modelling suite (2016–1 release) [74]. Runs were performed in the Desmond molecular dynamics software package using the OPLS_2005 force field through the Maestro interface (released with the Schrödinger suite) [75, 76]. The default Desmond relaxation protocol was used, featuring two rounds of minimization, followed by a series of gradually diminishing restraints over ~ 160 ps and a brief, 100 ps unrestrained segment at constant temperature and pressure of 300 K and 1.013 bar, respectively. Snapshots were aligned by Cα atoms and averaged over the last 100 ps to generate representative models. The minimized and relaxed active site models were then visualized in PyMOL [77].

### Molecular graphics

All molecular figures were generated using PyMOL [77].

## Additional file

Additional file 1: **Figure S1.** Average active site models for Ypd1-G68X variants. Representative active sites calculated by averaging the last 100 unrestrained ps following energy minimization and relaxation. G68X substitutions are labelled, depicted in red spheres. The phosphoryl group and conserved active site residues are depicted in stick representation. The first panel depicts the BeF_3_^−^ activated complex of Sln1-R1 and wild-type Ypd1 (PDB ID: 2R25) with BeF_3_^−^ depicted in stick representation. (TIF 4327 kb)

## Abbreviations

5-IAF: 5-iodoacetamidofluorescein
ATP: Adenosine Triphosphate
DHp: Dimerization and histidine phosphotransfer domain
FTIR: Fourier-transform infrared spectroscopy
GST: Glutathione S-transferase
HHK: Hybrid Histidine Kinase
HK: Histidine Kinase
HOG: High Osmolarity Glycerol
HPt: Histidine Phosphotransfer protein (or domain)
MAPK: Mitogen-Activated Protein Kinase
PEG: Polyethylene glycol
RMSD: Root Mean Square Deviation
RR: Response Regulator
Sln1-R1: Receiver domain of Sln1
Ssk1-R2: Receiver domain of Ssk1
