# Supplementary figures and images for: Role of the highly conserved G68 residue in the yeast phosphorelay protein Ypd1: implications for interactions between histidine phosphotransfer (HPt) and response regulator proteins
Source: BMC Biochem. 2019 Jan 21;20:1. doi: 10.1186/s12858-019-0104-5 (PMC6341664; doi:10.1186/s12858-019-0104-5)

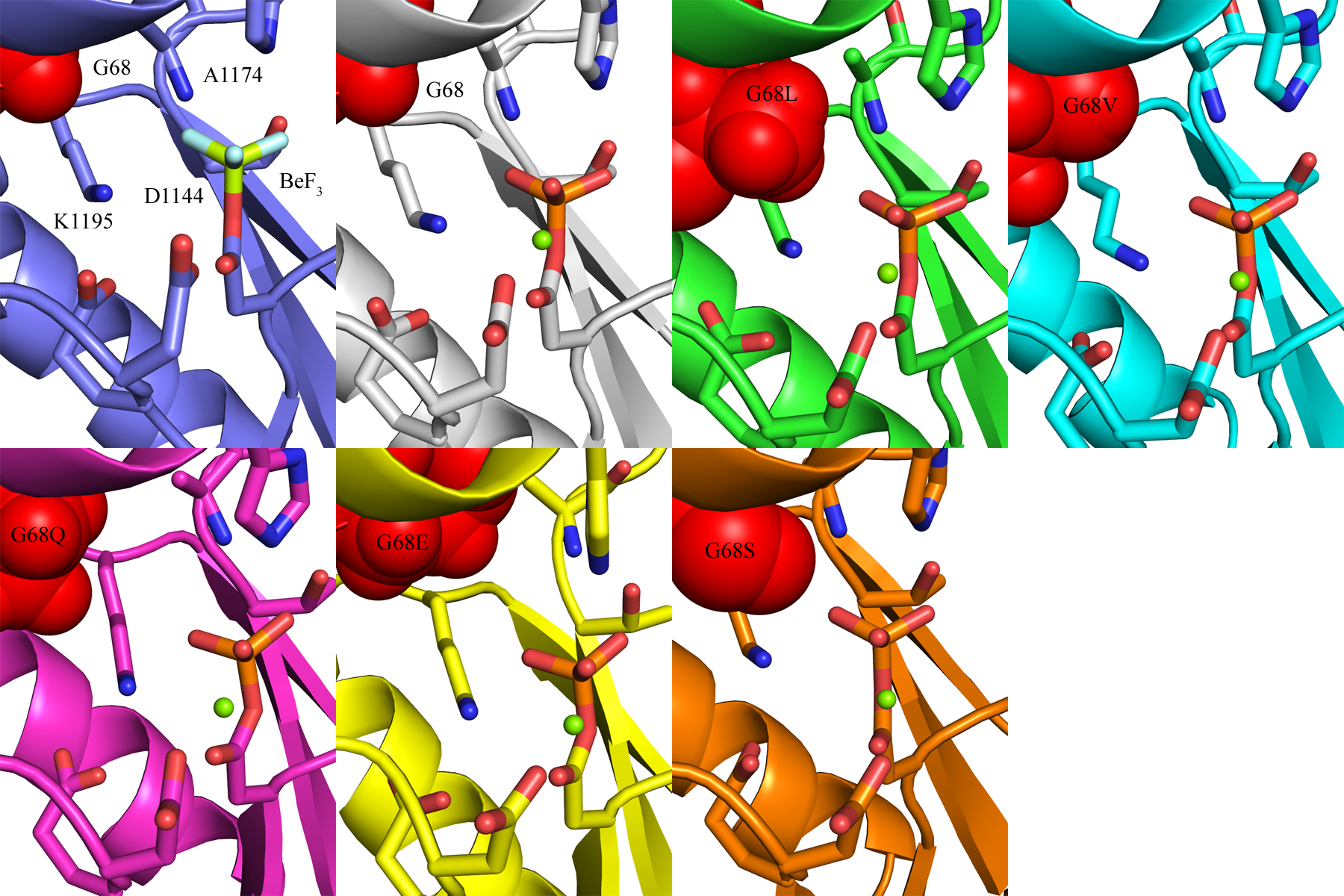

Supplement: Supplementary file 1 — Figure S1. Average active site models for Ypd1-G68X variants. Representative active sites calculated by averaging the last 100 unrestrained ps following energy minimization and relaxation. G68X substitutions are labelled, depicted in red spheres. The phosphoryl group and conserved active site residues are depicted in stick representation. The first panel depicts the BeF3− activated complex of Sln1-R1 and wild-type Ypd1 (PDB ID: 2R25) with BeF3− depicted in stick representation. (TIF 4327 kb) [file 12858_2019_104_MOESM1_ESM.tif]
